# Long-Term Follow-Up of Left Atrial Appendage Exclusion: Results of the V-CLIP Multi-Center Post-Market Study

**DOI:** 10.3390/jcm14155473

**Published:** 2025-08-04

**Authors:** Elias Zias, Katherine G. Phillips, Marc Gerdisch, Scott Johnson, Ahmed El-Eshmawi, Kenneth Saum, Michael Moront, Michael Kasten, Chanderdeep Singh, Gautam Bhatia, Hiroo Takayama, Ralph Damiano

**Affiliations:** 1 Department of Cardiothoracic Surgery, New York University Langone Medical Center, 530 1st Ave, Ste 9V, New York, NY 10016, USA; elias.zias@nyulangone.org; 2 Department of Cardiothoracic Surgery, Franciscan Alliance dba Franciscan Health Indianapolis, 8111 South Emerson Ave, Indianapolis, IN 46237, USA; mgerdisch@openheart.net; 3 Department of Cardiothoracic Surgery, Aurora Research Institute LLC, 2901 W Kinnickinnic River Pkwy, Ste 507, Milwaukee, WI 53215, USA; scott.johnson3@aah.org; 4 Department of Cardiothoracic Surgery, Icahn School of Medicine at Mount Sinai, 1 Gustave L. Levy Pl, New York, NY 10029, USA; ahmed.el-eshmawi@mountsinai.org; 5 Department of Cardiothoracic Surgery, Centra Lynchburg General Hospital, 1901 Tate Springs Rd, Lynchburg, VA 24501, USA; kenneth.saum@centrahealth.com; 6 Department of Cardiothoracic Surgery, Toledo Hospital, 1 ProMedica Parkway, Toledo, OH 43606, USA; morontmd@icloud.com; 7 Department of Cardiothoracic Surgery, Mercy Health Physicians, 1000 N Village Ave, Rockville Centre, NY 11570, USA; michael.kasten@stelizabeth.com; 8 Department of Cardiothoracic Surgery, Albany Medical Center, 43 New Scotland Ave, Albany, NY 12208, USA; chanderdeepsingh@guthrie.org; 9 Department of Cardiothoracic Surgery, Prisma Health-Greenville Memorial Hospital, 701 Grove Rd, Greenville, SC 29605, USA; gautam.bhatia@prismahealth.org; 10 Department of Cardiothoracic Surgery, Columbia University Medical Center, 630 W 168th St, New York, NY 10032, USA; ht2225@cumc.columbia.edu; 11 Department of Cardiothoracic Surgery, Washington University School of Medicine, 660 S Euclid Ave, St. Louis, MO 63110, USA; damianor@wustl.edu

**Keywords:** left atrial appendage, AtriClip, cardiac surgery

## Abstract

**Background**: Cardiac surgery patients with pre- or post-operative atrial fibrillation are at an increased risk for thromboembolic stroke, often due left atrial appendage (LAA) thrombus. Surgical LAA exclusion (LAAE) can be performed and must be complete to avoid increased thrombus formation. **Methods**: This prospective, multi-center, post-market study (NCT05101993) evaluated the long-term safety and performance of the epicardial V-shape AtriClip device. Patients ≥18 years who had received V-shape AtriClip devices during non-emergent cardiac surgery consented to a prospective 12-month follow-up visit and LAA imaging. The primary performance was LAAE without residual left atrium-LAA communication, assessed by imaging at the last follow-up visit. The primary safety was device- or implant procedure-related serious adverse events (SAEs) (death, major bleeding, surgical site infection, pericardial effusion requiring intervention, myocardial infarction) within 30 days. **Results**: Of 155 patients from 11 U.S. centers, 151 patients had evaluable imaging. Complete LAAE was obtained in all patients. Primary performance in the intent-to-treat population was met, with 97% (95% CI 93.52%, 99.29%; *p* = 0.0001) complete LAAE. Primary safety was met, with 100% (95% CI 97.75%, 100%; *p* < 0.0001) of patients free from pre-defined SAEs within 30 days. One device-related SAE was reported, which resolved intraprocedurally. Conclusions: AtriClip V-Clip showed safe and successful LAAE through 12 months of follow-up.

## 1. Introduction

Approximately 90% of thrombi in atrial fibrillation (AF) originate in the left atrial appendage (LAA) [1]. Surgical left atrial appendage exclusion (LAAE) has demonstrated significant efficacy in randomized trials such as LAAOS III, which showed a 33% relative reduction in stroke incidence over 3.8 years of follow-up [2].

Consensus guidelines from major international cardiology and surgical societies have given Class I recommendations to surgical LAAE during concomitant cardiac surgery in patients with AF [3,4,5,6]. However, as noted in the American College of Cardiology guidelines, a successful surgical LAAE technique and transesophageal echocardiography (TEE) imaging confirmation are important to ensure adequate LAA closure, evidenced by a lack of residual communication between the LA and LAA and a residual LAA neck of ≤10 mm [3]. Published evaluations of clinical outcomes after incomplete suture ligation during cardiac surgery in patients with AF have suggested the risks of thromboembolic events are greater when the LAA is incompletely closed than when it is left open [7,8].

Different surgical LAAE techniques have varying efficacies for closure. Suture ligation with either an encircling technique or running sutures was deemed ineffective in the LAAOS I feasibility trial, so this technique was excluded from the LAAOS II and III trials [9]. Other echocardiographic studies have also shown high incomplete closure rates with double layer sutures [10] and staples, with residual communication rates of 10–60% [11,12,13,14,15]. An epicardial clip (AtriClip) device has been evaluated in first-in-human, feasibility, and investigational device exemption (IDE) trials, as well as several observational, real-world studies. The device has been used in more than 500,000 patients worldwide, with consistently low major complication rates and high success in complete LAAE [16,17,18,19,20,21]. Clinical trials have evaluated the closed-ended, rectangular AtriClip (AOD1) [16,18], whereas published data on the V-shape clip (AOD2 or V-Clip) are limited. To this end, a retrospective–prospective, post-market study was performed to evaluate the long-term LAAE performance and safety of the V-Clip.

## 2. Methods

### 2.1. Patient Eligibility

The inclusion criteria were patients aged ≥18 years who had previously received an implantation of an AtriClip FLEX-V or PROV device during a non-emergent cardiac surgery and were willing to return for a scheduled follow-up visit and undergo either computed tomography angiography (CTA) or TEE imaging. Patients were excluded if they were unable, unwilling, or contraindicated to undergo TEE or CTA imaging, were pregnant or breastfeeding, or had active COVID-19 infection. To mitigate temporal bias of the AtriClip application experience, participating sites provided the Sponsor with a de-identified, anonymous list of potential study patients who had received an AtriClip study device within 10 to 24 months of the screening date. This listing was then randomized and returned to the site with the instruction to complete screening in the new sequence.

### 2.2. Study Design

**Figure 1:**
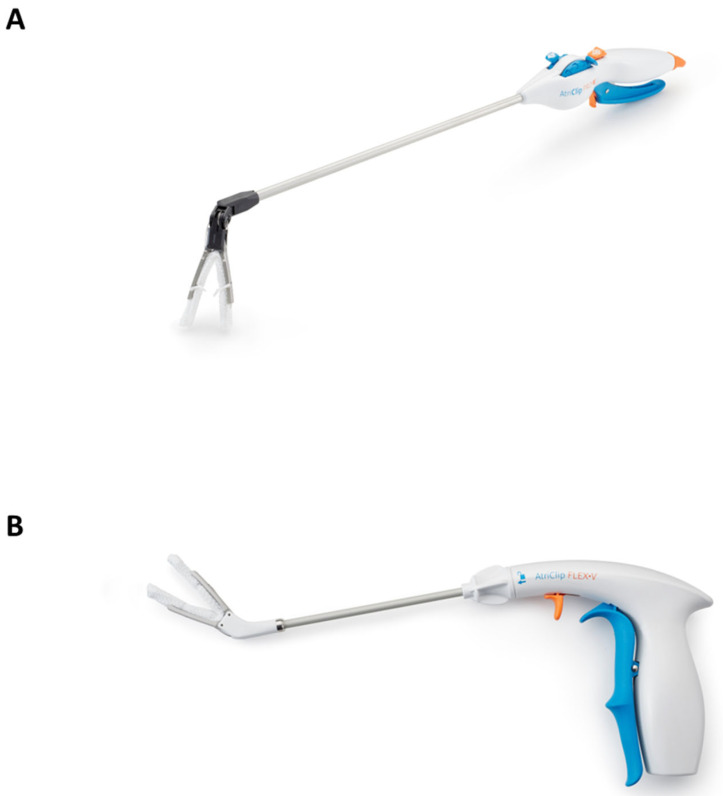
The AtriClip PROV (**A**) and FLEX-V (**B**) devices. The PROV and FLEX-V appliers have different features to accommodate surgeon preferences and approaches, but the V-shape implantable Clip (AOD2/V-Clip) is the same on each applier.

The Long-Term Follow-Up on LAA Exclusion using AtriClip (V-CLIP) study (NCT05101993) was a retrospective–prospective, multi-center, non-randomized, unblinded post-market study evaluating the long-term performance and safety of the AtriClip FLEX-V and PROV LAA Exclusion devices (AtriCure, Inc., Mason, OH, USA) for the exclusion of the LAA of the heart during concomitant cardiac procedures. The AtriClip FLEX-V and PROV models of the AtriClip LAA Exclusion System have a V-shape implantable clip (AOD2 or V-Clip) attached to a disposable applier (Figure 1). In the United States, the AtriClip LAA Exclusion system has FDA 510k-clearance and is indicated for the exclusion of the LAA, performed under direct visualization and in conjunction with other cardiac surgical procedures. Direct visualization in this context required that the surgeon was able to see the heart directly, with or without assistance from a camera, endoscope, or any other appropriate viewing technology.

The study design included retrospective and prospective data collection per the study visit schedule (Supplementary Table S1). Data at baseline, implant procedure, and 30 days post-implant procedure were collected retrospectively. All patients had previously received AtriClip implantation during a non-emergent cardiac surgery to be eligible for enrollment. Implantation of the AtriClip during open sternotomy and minimally invasive procedures has been well described in the literature [17]. CTA or TEE imaging at or after 12 months post-implantation was conducted prospectively and assessed independently by the core laboratory at New York University Langone Medical Center (New York, NY, USA) using a standardized imaging protocol. To help standardize the imaging across centers, the core laboratory developed CTA/TEE imaging protocols that participating sites were trained on during their site initiation visit.

### 2.3. Primary and Secondary Endpoints

The primary performance endpoint was LAAE, defined as the absence of residual communication between the LA and the LAA, as assessed by CTA or TEE imaging at the last follow-up visit. The performance goal (PG) for successful LAAE at least 12 months post-AtriClip implant was 80%, which was based on the range of 66–89.5% complete closure rates from published clinical literature on contemporary LAA closure devices [22].

The primary safety endpoint was the incidence of protocol-defined serious adverse events (SAEs) within 30 days of the implant procedure if related to the device and/or implant procedure, as adjudicated by an independent, non-investigator cardiac physician overseen by the independent contract research organization, Avania (San Diego, CA, USA). Protocol-defined SAEs included death, major bleeding (BARC3 and above), surgical site infection, pericardial effusion requiring intervention, and clinical diagnosis of myocardial infarction. The PG for primary safety was 10.5%. This rate accounted for a 1.5× margin of the upper confidence bound of complications within 45 days reported for a contemporary endocardial LAA occlusion device [22].

The secondary performance endpoint was residual LAA neck ≤ 10 mm, as assessed by CTA or imaging at the final 12-month visit. The secondary safety endpoint was device- or procedure-related SAEs through the final (12-month) visit.

### 2.4. Sample Size Calculation

Based on a PG of 80% successful LAAE and an expected success rate of 90%, a sample size of 143 patients would have provided 92% power using a one-sided Exact test, with α = 0.025 significance to demonstrate primary performance success. It was estimated that up to 170 patients would be needed for enrollment, assuming an attrition rate of 16% for the last follow-up visit. Based on the safety performance goal of 10.5% and an expected population MAE rate of up to 5%, a sample size of 170 patients would have 85.4% power using a one-sided Exact test, at α = 0.025 significance.

### 2.5. Statistical Analysis

For the primary performance endpoint of successful LAAE, an exact binomial test was conducted at a one-sided α = 0.025 level of significance to test the following hypothesis that the proportion of performance successes was significantly higher than the PG:

H_0_: *p* ≤ 80%

H_A_: *p* > 80%

where *p* was the responder rate at the last follow-up visit occurring at least 12 months post-procedure.

For the primary safety endpoint, an exact binomial test was conducted at a one-sided α = 0.05 level of significance to test the following hypothesis that the proportion of patients who had an SAE was significantly lower than the PG:

The hypothesis test for the safety endpoint was

H_0_: *q* ≥ 10.5%

H_A_: *q* < 10.5%

where *q* was the proportion of patients with SAEs through 30 days after AtriClip implantation.

Statistical analysis was performed by an independent contract research organization, Clintera LLC (Orange, NJ, USA), using SAS 9.4.

### 2.6. Role of the Sponsor and Funding Source

The study was sponsored and funded by AtriCure, Inc. The Sponsor participated in study design, execution, and manuscript writing under the direction of the authors.

## 3. Results

**Figure 2:**
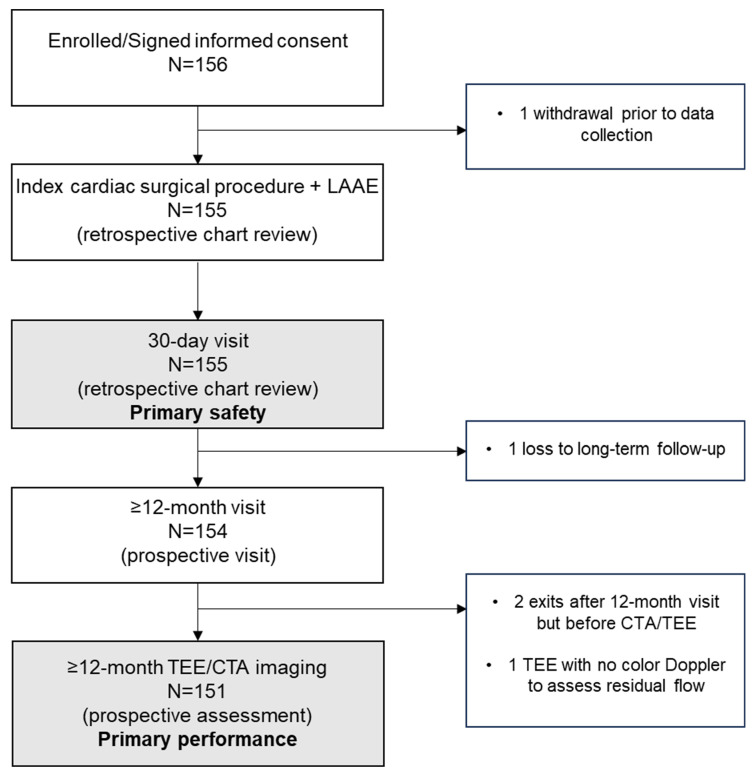
V-Clip post-market study attrition. CTA: computed tomography angiography; LAAE: left atrial appendage exclusion; TEE: transesophageal echocardiography.

A total of 156 patients from 11 sites in the United States were consented and enrolled in the study. The first and last patients were enrolled on 27 January 2022 and 23 November 2022, respectively. The median number of patients enrolled per site was 14 (9%). Study attrition is shown in Figure 2. One patient withdrew from the study prior to retrospective/prospective data entry; thus, the intention-to-treat (ITT) population consisted of 155 patients with an AtriClip implant. Due to being lost to follow-up (n = 1), a study exit prior to imaging acquisition (n = 2), and a lack of color Doppler used during TEE (n = 1), the modified ITT (mITT) population consisted of 151 patients with an AtriClip implant and CTA/TEE imaging allowing for the assessment of the primary performance endpoint (residual communication between the LA and LAA).

The retrospective baseline patient demographics and clinical characteristics of the ITT population are summarized in Table 1. The mean age was 65.8 years, mean BMI was 30 kg/m^2^, median CHA_2_DS_2_-VASc was 3, and median HAS-BLED was 2. Males accounted for 78% (121/155) of patients. Atrial fibrillation was reported to be present in 51% of patients at baseline. Medical history is shown in Supplemental Table S2. Of 154 patients with an available medical history, 71% had hypertension, 71% had hyperlipidemia, 48% had coronary artery disease, 44% had structural heart disease requiring surgery, and 23% had type II diabetes. Prior stroke and transient ischemic attack were reported in 7% and 6% of patients, respectively.

The cardiac surgical approaches were primarily sternotomy (57%; 88/155) and right mini-thoracotomy (19; 30/155) (Table 2). The primary cardiac surgeries during which the AtriClip was implanted are shown in Table 3. The five most frequent concomitant cardiac procedures (not mutually exclusive) included coronary artery bypass graft (36%; 55/155), AF surgery/ablation (33%; 51/155), mitral valve (33%; 51/155), aortic valve (17%; 27/155), and tricuspid valve (13%; 20/155). In total, 159 AtriClips were used in 155 patients. The PROV clip accounted for 46% (73/159) of AtriClip placements and FLEX-V accounted for 54% (86/159).

No implant procedure- or device-related primary SAE occurred within 30 days of the implant procedure (Table 4). Thus, the primary safety endpoint was met with 100% (155/155; 95% CI 97.75%, 100%) of patients being free from primary SAEs (*p* < 0.0001 compared to the safety goal).

In the ITT population, primary performance success of complete LAA exclusion with no residual flow between the LA and LAA was achieved in 97% (151/155; 95% CI 93.52%, 99.29%) of patients, which was significant compared to the PG (*p* = 0.0001) (Table 4). Although no residual flow was documented, three patients did not have imaging assessments available, and one patient did not have Doppler echo during TEE to evaluate residual communication. Thus, the primary performance success of 97% in the ITT population represents the most conservative assessment and assumes that those with missing data were failures. Among the 151 patients with evaluable imaging (mITT), complete LAAE was achieved in 100% (151/151; 95% CI 97.59%, 100%), exceeding the pre-specified performance goal of 80% (*p* < 0.0001).

A sub-analysis was completed to compare the primary performance success of complete LAA exclusion by the cardiac surgical approach (Table 5). The primary performance success of complete LAA exclusion was 100% (88/88; 95% CI 95.89%, 100%) for open sternotomy and 100% (63/63; 95% CI 94.3%, 100%) for minimally invasive surgery (MIS), both of which were significant compared to the PG (*p* = 0.0001). Primary performance success rates were 100% (33/33; 95% CI 89.4%, 100%) and 100% (30/30; 95% CI 88.43%, 100%) for minimally invasive left-sided thoracoscopy and minimally invasive right mini-thoracotomy, respectively, and were significant compared to the PG (MIS, left *p* = 0.0001; MIS, right *p* = 0.0012).

The secondary performance endpoint evaluated freedom from a residual neck (or stump) >10 mm. Of 152 patients with evaluable residual LAA neck data on imaging, 89% (135/152 patients) were free from a residual neck >10 mm (Table 6). Among the cases of residual neck > 10 mm, the applied clip was ACHV in 4 cases and PROV2 in 13 cases. There were no thrombi in residual stumps > 10 mm.

The secondary safety endpoint was the rate of device- or procedure-related SAEs through the last follow-up visit (12 months). One SAE (non-BARC3 bleeding) (0.6%; 1/155) was adjudicated as related to the device but resolved without sequalae. Four SAEs were deemed related to the general cardiac procedures but unrelated to the device or device implantation procedure. They were as follows: hemothorax requiring surgical intervention at seven days post-mitral valve and AF ablation via right mini-thoracotomy; pericardial effusion requiring surgical intervention at seven days post-AF ablation and staged hybrid convergent procedure via the left thoracoscopic approach; excessive bleeding (BARC3) requiring surgical intervention and transfusion during AF ablation via right mini-thoracotomy; and pericarditis requiring medication at 15 days post-aortic valve replacement via the upper mini J-sternotomy approach. These five SAEs occurred in cases in which PROV clips were used; however, as noted, only one case was adjudicated as related to the device. No unanticipated adverse events or unanticipated serious adverse device events were reported under the study protocol.

During the study follow-up, two strokes and two transient ischemic attacks occurred, which were unrelated to the device or implant procedure. None of the patients who experienced a stroke or transient ischemic attack had a residual stump >10 mm. No deaths occurred among the consenting study patients.

## 4. Discussion

The V-shape AtriClip (AOD2 or V-Clip) design provides physicians with another option for LAAE depending on the surgical approach, patient anatomy, and physician preference. Importantly, the principles behind the two AtriClip implant designs are the same. Both the AtriClip AOD1 and AOD2/V-Clip designs provide an atraumatic method for excluding the LAA from an epicardial approach and leverage dynamic, linear closure provided by the two parallel beams. The AOD2/V-Clip consists of a single piece of titanium, whereas the AOD1 clip has nitinol (a nickel-titanium alloy) springs that attach the two beams. Both clips are covered in a knit-braided polyester fabric to facilitate tissue ingrowth and enhance clip stability. Ultimately, the permanently implanted AtriClip cuts off the blood supply to the LAA, leading to controlled ischemic necrosis and cellular devitalization of the LAA tissue distal to the clip. The LAA tissue distal to the AtriClip subsequently atrophies and is resorbed [23,24,25]. Through pre-clinical animal studies and sensing/pacing exercises in human clinical cases, the AtriClip has also been shown to provide electrical isolation, which is relevant as the LAA is a known source of electrical activity in AF and is not electrically isolated by other appendage management devices [23,24,26,27].

In this post-market study, the AtriClip models with the V-Clip implant, FLEX-V and PROV, met both primary performance and safety endpoints, with high long-term success of complete LAAE (97.42% and 100% in ITT and mITT populations, respectively) evidenced by the lack of residual communication on TEE with color Doppler or CTA at 12 months of imaging. None of the five pre-specified device- or implant procedure-related SAEs (death, major bleeding, surgical site infection, pericardial effusion requiring intervention, and myocardial infarction) occurred within 30 days. Device-related SAEs through 12 months occurred at a low rate (0.6%). One non-BARC3 bleeding event occurred at the base of the LAA from the sutures during the surgical procedure, which was resolved with surgical glue and did not require transfusion. The other four secondary SAEs were related to the general surgical procedure. Nearly 89% of patients were free of a clinically significant residual neck.

Published studies utilizing the AtriClip AOD1 have shown comparable rates of safety and closure to those demonstrated for the AtriClip AOD2/V-Clip in the present study. The ATLAS feasibility trial, which included 370 patients who received AOD1 AtriClip implants, reported 98.9% intraoperative LAAE without residual communication or LAA neck > 10 mm in 370 patients who received AtriClip, with only one (0.3%) SAE related to the device/application. [18] The present study included patients who had placement of the AOD2 AtriClip through open sternotomy as well as through mini-thoracotomy and thoracoscopic ports, and it evaluated longer-term LAA exclusion at 12 months. Patients who received an AtriClip through both open sternotomy and minimally invasive surgeries had 100% complete LAAE. Caliskan et al. reported 100% complete LAAE with no residual communication and 97.7% freedom from residual neck > 10 mm in 43 patients who received the AtriClip through sternotomy and had a CTA at mean follow-up of 7.1 ± 0.8 years since the implant [28]. Kiankhooy et al. reported 100% LAAE without residual communication and 96% freedom from residual neck > 10 mm in 97 patients who received AtriClip through sternotomy/thoracotomy (n = 23) or video-assisted thoracoscopic surgery (VATS) (n = 74) and had a TEE at a mean of 1.87 years after the implant [21]. In a cohort of 65 patients who received totally thoracoscopic LAAE by AtriClip, Ellis et al. reported 94% LAAE free from residual LAA neck >10 mm by CTA at a mean of 2.82 years since the implant. [29] Recently, Ahmed et al. reported 81% of patients who received totally thoracoscopic AtriClip placement with hybrid convergent ablation were free from a residual stump > 10 mm by contrast-enhanced CT with 3–6 months post-procedure; no patient with a residual stump > 10 mm had a cerebrovascular accident during the 1-year follow-up [30]. As the authors noted, their residual stump rate was higher than in previously published studies. It was also higher than the residual neck rate found in the present V-Clip study. In the 2004 LAAOS I study, >10 mm residual stump was set as the threshold for insufficient occlusion because it represented >50% of the pre-occlusion length of the smallest appendage in that study [9]. The internal LAA neck may also be smooth rather than trabeculated up to this point, though this is variable. While it is logical that a significant residual LAA neck could serve as a site of thrombus formation and residual necks > 10 mm are conventionally considered suboptimal, recent findings suggest that the correlation with thromboembolic events may not be linear, necessitating further prospective evaluation [30]. Furthermore, residual LAA neck measurements can be subject to inter-operator variability and patient positioning. For those reasons, the primary performance endpoint of the V-Clip study was residual communication between the LA and LAA, with the residual LAA neck as a secondary endpoint.

This study was not designed or powered to evaluate the association between LAA exclusion and thromboembolic events, and some patients had AF ablation. However, two strokes (one ischemic and one cardioembolic) and two transient ischemic attacks were reported in the study period in patients who had residual LAA necks < 10 mm. The strokes occurred 104 and 824 days post-implant procedure. Both patients who experienced a stroke were on anticoagulants at the 12-month follow-up, but there had been a change in anticoagulation medication from the baseline to the 12-month visit. The transient ischemic attacks occurred at 17 and 27 days post-implant procedure, and both patients had subtherapeutic international normalized ratios. One patient was on anticoagulants at baseline and the 12-month follow-up with no change in medication, while the other patient was not on any anticoagulants at any time. The stroke rate in the ITT patient population was 1% (2/155). The reported peri- and postoperative stroke rates for patients undergoing common cardiac procedures is 1.0–5.0% [31,32]. Based on a median CHA_2_DS_2_-VASc of three in this study population, the estimated annual stroke risk was 3.2%.

Transesophageal echocardiography assessments using color Doppler and four mid-esophageal views (shallow-, mid-, steep-angles, and, if possible, 3D views) at baseline and intra-procedurally, before and after clip deployment, are critical to successful LAAE with AtriClip. The baseline assessment allows for the evaluation of the LAA anatomy and morphology and provides confirmation that the circumflex artery is patent and there is no thrombus in the LAA. Intra-procedural imaging after AtriClip placement (during beating heart surgery), but before its deployment from the applier, allows for repositioning of the clip if needed to ensure proper closure and confirms that adjacent structures are not affected. Post-deployment imaging should also be performed with findings documented.

Taken together, the long-term exclusion data on both AtriClip implant designs exceeds the performance of other surgical methods, such as sutures and staples, which have yielded incomplete closure rates from 10% to 60% [7,8,9,10,11,12,13,14,15]. Peri-device leaks (PDL) after endocardial LAA occlusion with percutaneous devices have also been observed, with the Amulet IDE reporting PDL (>0 mm) rates of 37% and 54% for the Amulet and Watchman 2.5 devices at 45 days post-procedure, respectively, and 37% and 47% at 12 months, respectively [33]. While PDL ≤ 5 mm have generally been considered acceptable for endocardial occlusion devices, a long-term data analysis from >1000 patients in WATCHMAN trials reported that even patients who had PDL ≤ 5 mm at the 1-year follow-up had nearly twice the risk of ischemic stroke (most non-disabling) and systemic embolism than patients who did not have PDL [34]. In that analysis, 28% of patients had PDL. In the V-Clip study, 100% of patients who had evaluable color Doppler TEE or CTA 12 months after the AtriClip implant had no residual communication between the LA and LAA.

One limitation of the V-Clip study is the retrospective component of the trial design, which could have led to selection bias. However, as described in the methods, randomization of the treated patients at each site was carried out prior to screening to mitigate temporal bias. The study inclusion criteria required patients to return for 12-month follow-up imaging, which could have introduced a healthy survivor or selection bias. Therefore, patients with complications or limited follow-up may be underrepresented. Women and racial/ethnic minorities represented smaller proportions of the patient enrollment. The study design was non-randomized; thus, the absence of a control group precludes direct comparative effectiveness or safety conclusions. Additionally, the study was not designed or powered to evaluate whether there was an association between residual communication or LAA neck and thromboembolic events.

In summary, this study demonstrates that the V-Clip AtriClip device offers highly effective and safe long-term LAAE, supporting its use as a standard adjunct during cardiac surgery. The LAAE rates achieved with no evidence of residual communication (leaks) are substantially higher than those reported for other surgical LAA closure methods and endocardial occlusion devices, which is consistent with prior published evidence on AtriClip. Two ongoing, IDE clinical trials (NCT03732794, NCT05478304) include the use of the FLEX-V AtriClip and will provide additional prospective evidence of closure performance and safety.

## Figures and Tables

**Table 1:** Baseline clinical characteristics and demographics.

Characteristic	Result
Age, years	N = 155
Mean	65.8
Median	66.0
Range	41, 85
Sex, % (n)	N = 155
Male	78.1 (121)
Female	21.9 (34)
Race, % (n)	N = 155
White	94.2 (146)
Black	3.2 (5)
Asian	1.3 (2)
Other	1.3 (2)
Ethnicity, % (n)	N = 155
Not Hispanic/Latino	99.4 (154)
Hispanic/Latino	0.6 (1)
BMI, kg/m^2^	N = 152
Mean	30.0
Median	29.0
Range	18.7, 51.4
Atrial arrhythmia, % (n)	N = 155
Paroxysmal AF	22.6 (35)
Persistent AF	16.8 (26)
Longstanding persistent AF	11.6 (18)
Not reported ^1^	49 (76)
NYHA Classification, % (n)	N = 155
I	9.7 (15)
II	20.0 (31)
III	9.7 (15)
IV	1.3 (2)
No heart failure	12.9 (20)
Not assessed ^1^	46.5 (72)
CHA_2_DS_2_-VASc, % (n)	N = 155
Mean	2.7
Median	3
0	3.2 (5)
1	18.1 (28)
2	23.2 (36)
3	27.1 (42)
4	13.5 (21)
5	6.5 (10)
6	3.2 (5)
7	0.6 (1)
Not done	4.5 (7)
HAS-BLED, % (n)	N = 155
Mean	2.0
Median	2
0	6.5 (10)
1	14.2 (22)
2	25.8 (40)
3	17.4 (27)
4	6.5 (10)
5	1.3 (2)
6	0.0 (0)
7	0.0 (0)
8	0.0 (0)
9	0.0 (0)
Not done	28.4 (44)

^1^ Not reported and not assessed were selection choices on the case report form and do not indicate missing data. AF: atrial fibrillation; BMI: body mass index; NYHA: New York Heart Association

**Table 2:** Cardiac surgical approaches.

Surgical Approach (N = 155)	% (n)
Sternotomy	58.1 (90)
Minimally Invasive, Right Mini-Thoracotomy	20.6 (32)
Minimally Invasive, Left-Sided Thoracoscopy	21.3 (33)

**Table 3:** Concomitant Surgical Procedure.

Concomitant Surgical Procedure (N = 155)	% (n)
Coronary artery bypass graft	35.5 (55)
AF ablation/surgery	34.8 (54)
Mitral valve	32.9 (51)
Aortic valve	17.4 (27)
Tricuspid valve	12.9 (20)
Convergent hybrid MAZE (various)	11.0 (17)
MAZE (various)	5.8 (9)
Patent foramen ovale closure	4.5 (7)
Aortic surgery	3.9 (6)
Atrial septal defect repair	1.3 (2)
Aortic aneurysmectomy	0.6 (1)
Excision of atrial myxoma	0.6 (1)
Extended septal myectomy	0.6 (1)
Extended septal myectomy, ascending aorta replacement	0.6 (1)
Left atrial appendage closure ꭞ	0.6 (1)
Partial atrial ventricular canal closure	0.6 (1)
Pulmonary vein isolation	0.6 (1)

ꭞ Stand-alone LAA closure procedure reported.

**Table 4:** Primary safety and performance endpoints.

Endpoint	% (95% Confidence Interval); n/N	*p*-Value ^a^
Primary Safety: Freedom from primary SAE within 30 days of the implant procedure ^b^	100 (97.75, 100); 155/155	<0.0001
Primary Performance (ITT population): LAA exclusion with no residual communication between the LA and LAA ^c^	97.42 (93.52, 99.29); 151/155	0.0001
Primary Performance (mITT population): LAA exclusion with no residual communication between the LA and LAA	100 (97.59, 100); 151/151	0.0001

^a^ *p*-values are based on an exact binomial analysis comparing the endpoint result to the performance goal. ^b^ Primary SAEs were defined in the protocol as death, major bleeding, surgical site infection, pericardial effusion requiring intervention, and myocardial infarction related to the device and/or implanted procedure, as adjudicated by an independent medical monitor. ^c^ Of the four patients who failed the primary performance endpoint, three patients did not have available imaging data and one patient did not have color Doppler during imaging for flow evaluation. Imaging was evaluated by an independent core laboratory. LA: left atrium; LAA: left atrial appendage; ITT: intention-to-treat; mITT: modified ITT; SAEs: serious adverse events.

**Table 5:** Sub-analysis of primary performance endpoints by cardiac surgical procedure.

Sub-Analysis	Performance Endpoint Rate, % (95% Confidence Interval); n/N	*p*-Value ^a^
Open sternotomy	100 (95.89, 100); 88/88	0.0001
Minimally Invasive Surgery	100 (94.3, 100); 63/63	0.0001
Left access	100 (89.4, 100); 33/33	0.0001
Right access	100 (88.43, 100); 30/30	0.0012

^a^ *p*-values are based on an exact binomial analysis comparing the endpoint result to the performance goal.

**Table 6:** Secondary performance and safety endpoints.

Endpoints	% (n/N)
Secondary Performance: Residual LAA neck ≤ 10 mm at last follow-up visit ^a^	88.8 (135/152)
Secondary Safety: Device- or procedure-related SAEs through last (12-month) follow-up visit ^b^	3.22 (5/155)

^a^ 152 out of 155 patients in the study had evaluable imaging for residual neck. ^b^ These SAEs were (1) bleeding (non-BARC3) during atrial fibrillation (AF) ablation via a subxiphoid approach that required intervention with surgical glue (related to AtriClip); (2) hemothorax requiring surgical intervention at 7 days post-mitral valve and AF ablation via right mini-thoracotomy (related to general surgical procedure); (3) pericardial effusion requiring surgical intervention at 7 days post-AF ablation and staged hybrid convergent procedure via left thoracoscopic approach (related to general surgical procedure); (4) excessive bleeding (BARC3) requiring surgical intervention and transfusion during AF ablation via right mini-thoracotomy (related to general surgical procedure); and (5) pericarditis requiring medication at 15 days post-aortic valve via the upper mini J-sternotomy approach (related to general surgical procedure). LAA: let atrial appendage; SAE: serious adverse events.

## Data Availability

The original contributions presented in this study are included in the article/Supplementary Material. Further inquiries can be directed to the corresponding author(s).

## Supplementary Materials

The following supporting information can be downloaded at: https://www.mdpi.com/article/10.3390/jcm14155473/s1, Table S1: V-Clip study visit schedule. AE: adverse event; BMI: body mass index; BP: blood pressure; CTA: computed tomography angiography; NYHA: New York Heart Association; SAE: serious adverse event; TEE: transesophageal echocardiography; Table S2: Patient Medical History.
