# Supplementary material for: Long-Term Follow-Up of Left Atrial Appendage Exclusion: Results of the V-CLIP Multi-Center Post-Market Study
Source: J Clin Med. 2025 Aug 4;14(15):5473. doi: 10.3390/jcm14155473 (PMC12346947; doi:10.3390/jcm14155473)
Supplement: Supplementary file 1 [file jcm-14-05473-s001.zip › jcm-3687381-supplementary.pdf]

Supplemental Table S1. V-Clip study visit schedule. AE: adverse event; BMI: body mass index; BP: blood pressure; CTA: computed tomography angiography; NYHA: New York Heart Association; SAE: serious adverse event; TEE: transesophageal echocardiography

**Trial Activity**	**Visit 1**	**Visit 2**	**Visit 3**	**Visit 4**
	**Baseline (Within 30 days of index procedure)**	**Index Procedure**	**30-days Post-Procedure (Within 23-37 days of index procedure)**	**12-months Post-Procedure (≥ 12-months from index procedure)**
Informed Consent & Inclusion/Exclusion	**X**			
Demographics (Age, Sex, Race, Ethnicity)	**X**			
Vital Signs (BMI, BP, Heart Rate)	**X**			**X**
Medical/Surgical/Cardiac History ($CHADS_2$, CHA_2DS_2-VASc, HAS-BLED scores)	**X**			
NYHA Classification	**X**		**X**	**X**
Intra-Operative TEE, if done		**X**		
CTA or TEE Imaging				**X**
Cardiac Medications/Adjustment Review	**X**			**X**
Adverse Events (AEs/SAEs)		**X**	**X**	**X**
Surgical Procedure		**X**		

Supplemental Table S2. Patient Medical History

Characteristics	[% (n/N)]
Hypertension	71.4% (110/154)
Hyperlipidemia	70.8% (109/154)
Coronary Artery Disease	48.1% (74/154)
Structural Heart Disease requiring surgery	43.5% (67/154)
Diabetes Mellitus - Type II	23.4% (36/154)
Atrial Fibrillation - Paroxysmal	22.7% (35/154)
Congestive Heart Failure (CHF)	20.8% (32/154)
Cancer	18.2% (28/154)
Atrial Fibrillation - Persistent	16.9% (26/154)
Obstructive Sleep Apnea	12.3% (19/154)
Atrial Fibrillation - Longstanding Persistent	11.7% (18/154)
Myocardial Infarction	11.0% (17/154)
Cardiomyopathy	10.4% (16/154)
Benign prostatic hyperplasia	8.4% (13/154)
Obstructive Sleep Apnea	7.1% (11/154)
Peripheral Vascular Disease	7.1% (11/154)
Stroke - Ischemic	7.1% (11/154)
Anxiety	6.5% (10/154)
Gastroesophageal reflux disease	5.8% (9/154)
Transient Ischemic Attack	5.8% (9/154)
Gout	5.2% (8/154)
Nephrolithiasis	3.9% (6/154)
Hypothyroidism	3.2% (5/154)
Mitral Valve Regurgitation	3.2% (5/154)
Obesity	3.2% (5/154)
Bleeding Diathesis or Suspected Coagulopathy	2.6% (4/154)
Cardiac Murmur	2.6% (4/154)
Gastroesophageal Reflux Disease	2.6% (4/154)
Osteoarthritis	2.6% (4/154)
Presence of Implantable Cardiac Device - Pacemaker	2.6% (4/154)
Anemia	1.9% (3/154)
Asthma	1.9% (3/154)
Chronic kidney disease	1.9% (3/154)
Deep Vein Thrombosis	1.9% (3/154)
Diabetes Mellitus - Type I	1.9% (3/154)
Erectile Dysfunction	1.9% (3/154)
ETOH Abuse	1.9% (3/154)
Hyperthyroidism	1.9% (3/154)

Supplemental Table S2. Patient Medical History

Characteristics	[% (n/N)]
Presence of Implantable Cardiac Device - ILR	1.9% (3/154)
Rheumatic Heart Disease	1.9% (3/154)
Stroke - Hemorrhagic	1.9% (3/154)
Vertigo	1.9% (3/154)
Vitamin D deficiency	1.9% (3/154)
Abdominal Aortic Aneurysm	1.3% (2/154)
Aortic aneurysm	1.3% (2/154)
Aortic Stenosis	1.3% (2/154)
Arteriosclerotic heart disease	1.3% (2/154)
Blood Dyscrasia	1.3% (2/154)
Carotid artery stenosis	1.3% (2/154)
Cervical Degenerative Disc	1.3% (2/154)
Cervical Spine Stenosis	1.3% (2/154)
Cholecystitis	1.3% (2/154)
Chronic Diastolic Heart Failure	1.3% (2/154)
Chronic Obstructive Pulmonary Disease (FEV1/FVC/<70%)	1.3% (2/154)
Colon Polyps	1.3% (2/154)
Congenital Heart Defect	1.3% (2/154)
Degenerative Joint Disease	1.3% (2/154)
Depression	1.3% (2/154)
Diverticulitis	1.3% (2/154)
Diverticulosis	1.3% (2/154)
Erectile Dysfunction	1.3% (2/154)
Gastric Bypass Surgery	1.3% (2/154)
Hyperglycemia	1.3% (2/154)
Hypotension	1.3% (2/154)
Impaired fasting glucose	1.3% (2/154)
Kidney Stone	1.3% (2/154)
Low Back Pain	1.3% (2/154)
Macular Degeneration	1.3% (2/154)
Migraines	1.3% (2/154)
Mild Tricuspid Valve Regurgitation	1.3% (2/154)
Morbid Obesity	1.3% (2/154)
Normal pressure hydrocephalus	1.3% (2/154)
Osteoarthritis Hip	1.3% (2/154)
Pleural Effusion	1.3% (2/154)
Pneumonia	1.3% (2/154)
Pulmonary Hypertension	1.3% (2/154)

Supplemental Table S2. Patient Medical History

Characteristics	[% (n/N)]
Right Bundle Branch Block	1.3% (2/154)
Sarcoidosis	1.3% (2/154)
Small bowel obstruction	1.3% (2/154)
Spinal Stenosis	1.3% (2/154)
Ulcerative Colitis	1.3% (2/154)
Unstable Angina	1.3% (2/154)
Acid Reflux	0.6% (1/154)
Active Infection	0.6% (1/154)
Acute blood loss anemia	0.6% (1/154)
Acute Hypoxemic Respiratory Failure	0.6% (1/154)
Acute Kidney Injury	0.6% (1/154)
Adenoma Colon	0.6% (1/154)
Attention-deficit/hyperactivity disorder	0.6% (1/154)
Allergic Rhinitis	0.6% (1/154)
Amaurosis Fugax	0.6% (1/154)
Anal Fissure	0.6% (1/154)
Aortic Ectasia	0.6% (1/154)
Aortic Plaque	0.6% (1/154)
Aortic valve disorder	0.6% (1/154)
Apical ballooning syndrome	0.6% (1/154)
Appendicitis	0.6% (1/154)
Ascending Aortic Aneurysm	0.6% (1/154)
Atrial Septal Defect Closure	0.6% (1/154)
Atrial Flutter	0.6% (1/154)
Atrial Myxoma	0.6% (1/154)
Atrial Tachycardia	0.6% (1/154)
Atrioventricular Node Dysfunction	0.6% (1/154)
Attention deficit Disorder Disease	0.6% (1/154)
Autologous bone marrow transplantation status	0.6% (1/154)
Axillary Abscess	0.6% (1/154)
Back pain	0.6% (1/154)
Barrett’s Esophagus	0.6% (1/154)
Benign hypertensive heart disease	0.6% (1/154)
Benign Neoplasm Neck	0.6% (1/154)
Bilateral carotid stenosis	0.6% (1/154)
Bilateral Cataracts	0.6% (1/154)
Bilateral Knee Replacement	0.6% (1/154)
Bradycardia	0.6% (1/154)

Supplemental Table S2. Patient Medical History

Characteristics	[% (n/N)]
Breast Lumpectomy	0.6% (1/154)
Bronchitis	0.6% (1/154)
Bulging Cervical Disc	0.6% (1/154)
Bursitis Right Shoulder	0.6% (1/154)
Cardiac arrest	0.6% (1/154)
Carpal Tunnel Release	0.6% (1/154)
Cataracts	0.6% (1/154)
Cellulitis Left Lower Extremity	0.6% (1/154)
Cerebral aneurysm	0.6% (1/154)
Cervical Spine Surgery	0.6% (1/154)
Cervical Spondylosis	0.6% (1/154)
Chewing Tobacco Use	0.6% (1/154)
Cholelithiasis	0.6% (1/154)
Chronic Diarrhea	0.6% (1/154)
Chronic Maxillary Sinusitis	0.6% (1/154)
Chronic Otitis Media	0.6% (1/154)
Chronic Pain - Right Arm	0.6% (1/154)
Chronic Sinusitis	0.6% (1/154)
Chronic Venous Insufficiency	0.6% (1/154)
Chronic Venous Stasis	0.6% (1/154)
Chronic Venous Stasis w/ cellulitis	0.6% (1/154)
Colon Polyp	0.6% (1/154)
Complete Heart Block	0.6% (1/154)
Complete Rupture Rotator Cuff	0.6% (1/154)
COPD (chronic obstructive pulmonary disease)	0.6% (1/154)
Crohn's Disease	0.6% (1/154)
Deep Vein Thrombosis - Right Leg	0.6% (1/154)
Deviated Nasal Septum	0.6% (1/154)
Deviated Septum	0.6% (1/154)
Diabetic retinopathy	0.6% (1/154)
Difficult Intubation	0.6% (1/154)
Diffuse Idiopathic skeletal hyperostosis	0.6% (1/154)
Duodenal ulcers	0.6% (1/154)
Dyslipidemia	0.6% (1/154)
Dyspnea	0.6% (1/154)
Dyspnea on Exertion	0.6% (1/154)
Eczematous Dermatitis of Eyelid	0.6% (1/154)
Endocarditis	0.6% (1/154)

Supplemental Table S2. Patient Medical History

Characteristics	[% (n/N)]
Enlarged Aortic Root	0.6% (1/154)
Erosion in Gastric Antrum	0.6% (1/154)
Erosive Esophagitis	0.6% (1/154)
Esophageal Reflux	0.6% (1/154)
Esophageal Stricture	0.6% (1/154)
Factor V Leiden	0.6% (1/154)
Fibroadenoma Breast	0.6% (1/154)
Fibromyalgia	0.6% (1/154)
Gastroesophageal Reflux	0.6% (1/154)
GI AV malformation s/p argon laser	0.6% (1/154)
Graft versus host disease	0.6% (1/154)
H. pylori infection	0.6% (1/154)
Hand Laceration	0.6% (1/154)
Headaches	0.6% (1/154)
Heart Failure Preserved Ejection Fraction	0.6% (1/154)
Hematoma Adrenal Gland	0.6% (1/154)
Hemorrhoids	0.6% (1/154)
Hepatic Insufficiency	0.6% (1/154)
Hernia	0.6% (1/154)
Herniorrhaphy	0.6% (1/154)
Hypercholesteremia	0.6% (1/154)
Hyperuricemia	0.6% (1/154)
Hyponatremia	0.6% (1/154)
Impotence	0.6% (1/154)
Incarcerated Ventral Hernia	0.6% (1/154)
Insulin Resistance	0.6% (1/154)
Internal Hemorrhoids	0.6% (1/154)
Intervertebral disc disorder	0.6% (1/154)
Kidney Laceration	0.6% (1/154)
Knee Replacement	0.6% (1/154)
L2 Compression Fracture	0.6% (1/154)
Leaking Pancreas	0.6% (1/154)
Left Bundle Branch Block	0.6% (1/154)
Left carotid artery stenosis	0.6% (1/154)
Left Knee Arthritis	0.6% (1/154)
Left Knee Pain	0.6% (1/154)
Left Lower Lobe Pneumonia	0.6% (1/154)
Left Ovarian Torsion	0.6% (1/154)

Supplemental Table S2. Patient Medical History

Characteristics	[% (n/N)]
Liver Cirrhosis	0.6% (1/154)
Liver Laceration	0.6% (1/154)
Lower GI and rectal bleed	0.6% (1/154)
Lumbar disc displacement	0.6% (1/154)
Lumbar Fracture	0.6% (1/154)
Left Ventricular Dysfunction	0.6% (1/154)
Lyme disease	0.6% (1/154)
Melanoma	0.6% (1/154)
Metabolic syndrome	0.6% (1/154)
Migraine	0.6% (1/154)
Mitral Valve disease	0.6% (1/154)
Mitral Valve Prolapse	0.6% (1/154)
Methicillin-resistant Staphylococcus aureus	0.6% (1/154)
Multiple Dental Abscesses	0.6% (1/154)
Multiple Rib Fractures	0.6% (1/154)
Multiple sclerosis	0.6% (1/154)
Neck pain	0.6% (1/154)
Nephrolithiasis	0.6% (1/154)
Nonrheumatic MV Prolapse	0.6% (1/154)
Non-sustained Ventricular Tachycardia	0.6% (1/154)
Osteoarthritis of left shoulder	0.6% (1/154)
Osteoarthritis of right hip	0.6% (1/154)
Osteoporosis	0.6% (1/154)
Palpitations	0.6% (1/154)
Pancreatitis	0.6% (1/154)
Penile Psoriasis	0.6% (1/154)
Plantar Fasciitis	0.6% (1/154)
Pneumothorax	0.6% (1/154)
Poor Circulation	0.6% (1/154)
Post Operative Nausea and Vomiting	0.6% (1/154)
Post-Traumatic Stress Disorder	0.6% (1/154)
Prediabetes	0.6% (1/154)
Premature Ventricular Contractions	0.6% (1/154)
Presence of Implantable Cardiac Device - ICD	0.6% (1/154)
Psoriasis	0.6% (1/154)
Pulmonary Edema	0.6% (1/154)
Pulmonary embolism	0.6% (1/154)
Pulmonary Nodule	0.6% (1/154)

Supplemental Table S2. Patient Medical History

Characteristics	[% (n/N)]
Pulmonary nodules	0.6% (1/154)
Pulmonary Vein Isolation	0.6% (1/154)
Premature Ventricular Contraction Ablation	0.6% (1/154)
Premature Ventricular Contraction	0.6% (1/154)
Remote Lung Infarction	0.6% (1/154)
Renal Failure and/or dialysis	0.6% (1/154)
Renal Lesion	0.6% (1/154)
Restless Leg Syndrome	0.6% (1/154)
Retinal Detachment	0.6% (1/154)
Retinal Vein Occlusion	0.6% (1/154)
Rheumatic Aortic Stenosis	0.6% (1/154)
Rheumatoid arthritis	0.6% (1/154)
Rib Fracture	0.6% (1/154)
Right Carotid artery occlusion	0.6% (1/154)
Right greater saphenous vein thrombosis	0.6% (1/154)
Right Shoulder Surgery	0.6% (1/154)
Right Ventricular Dilation	0.6% (1/154)
Right ventricular systolic dysfunction	0.6% (1/154)
Seizure	0.6% (1/154)
Sepsis	0.6% (1/154)
Severe aortic stenosis	0.6% (1/154)
Severe symptomatic non-rheumatic aortic stenosis	0.6% (1/154)
Sick Sinus Syndrome	0.6% (1/154)
Sinus Bradycardia	0.6% (1/154)
Sixth nerve palsy	0.6% (1/154)
Sleep Apnea	0.6% (1/154)
Solid food dysphagia	0.6% (1/154)
Spinal Stenosis L4-5	0.6% (1/154)
Stasis dermatitis	0.6% (1/154)
Statin Intolerance	0.6% (1/154)
Stenosis of carotid artery	0.6% (1/154)
Stent placed due to coronary artery disease	0.6% (1/154)
Subclavian vein stenosis	0.6% (1/154)
Tear medial meniscus	0.6% (1/154)
Thoracic aortic aneurysm	0.6% (1/154)
Thromboembolism	0.6% (1/154)
Thyroid disease	0.6% (1/154)
Tremors	0.6% (1/154)

Supplemental Table S2. Patient Medical History

Characteristics	[% (n/N)]
Tremors-fingers	0.6% (1/154)
Tricuspid Insufficiency	0.6% (1/154)
Tricuspid valve disease	0.6% (1/154)
Trigeminal neuralgia	0.6% (1/154)
Tubular Adenoma	0.6% (1/154)
Tubular adenoma of colon	0.6% (1/154)
Ulcerative Colitis	0.6% (1/154)
Umbilical Hernia	0.6% (1/154)
Urine retention	0.6% (1/154)
Varicose Veins	0.6% (1/154)
Vasovagal syncope	0.6% (1/154)
Ventricular Fibrillation	0.6% (1/154)
Ventricular Tachycardia	0.6% (1/154)
Vertebral Osteomyelitis	0.6% (1/154)
Vision loss right eye	0.6% (1/154)
Vitamin B 12 Deficiency	0.6% (1/154)
